# Dynamics of Soil Bacterial and Fungal Communities During the Secondary Succession Following Swidden Agriculture IN Lowland Forests

**DOI:** 10.3389/fmicb.2021.676251

**Published:** 2021-06-07

**Authors:** Qiang Lin, Petr Baldrian, Lingjuan Li, Vojtech Novotny, Petr Heděnec, Jaroslav Kukla, Ruma Umari, Lenka Meszárošová, Jan Frouz

**Affiliations:** ^1^Biology Centre of the Czech Academy of Sciences, Institute of Soil Biology and SoWa Research Infrastructure, České Budějovice, Czechia; ^2^Faculty of Science, Institute for Environmental Studies, Charles University, Praha, Czechia; ^3^Laboratory of Environmental Microbiology, Institute of Microbiology of the CAS, Praha, Czechia; ^4^Institute of Entomology, Biology Centre of the Czech Academy of Sciences and University of South Bohemia, České Budějovice, Czechia; ^5^New Guinea Binatang Research Center, Madang, Papua New Guinea; ^6^Department of Geosciences and Natural Resource Management, Faculty of Science, University of Copenhagen, Frederiksberg, Denmark; ^7^Engineering Research Center of Soil Remediation of Fujian Province University, College of Resources and Environment, Fujian Agriculture and Forestry University, Fuzhou, China

**Keywords:** ecological succession, slash-and-burn, rare bacteria and fungi, tropical forests, soil microbiome

## Abstract

Elucidating dynamics of soil microbial communities after disturbance is crucial for understanding ecosystem restoration and sustainability. However, despite the widespread practice of swidden agriculture in tropical forests, knowledge about microbial community succession in this system is limited. Here, amplicon sequencing was used to investigate effects of soil ages (spanning at least 60 years) after disturbance, geographic distance (from 0.1 to 10 km) and edaphic property gradients (soil pH, conductivity, C, N, P, Ca, Mg, and K), on soil bacterial and fungal communities along a chronosequence of sites representing the spontaneous succession following swidden agriculture in lowland forests in Papua New Guinea. During succession, bacterial communities (OTU level) as well as its abundant (OTU with relative abundance > 0.5%) and rare (<0.05%) subcommunities, showed less variation but more stage-dependent patterns than those of fungi. Fungal community dynamics were significantly associated only with geographic distance, whereas bacterial community dynamics were significantly associated with edaphic factors and geographic distance. During succession, more OTUs were consistently abundant (*n* = 12) or rare (*n* = 653) for bacteria than fungi (abundant = 6, rare = 5), indicating bacteria were more tolerant than fungi to environmental gradients. Rare taxa showed higher successional dynamics than abundant taxa, and rare bacteria (mainly from Actinobacteria, Proteobacteria, Acidobacteria, and Verrucomicrobia) largely accounted for bacterial community development and niche differentiation during succession.

## Introduction

Slash-and-burn is a widespread agricultural technique in tropical forests (Whitfeld et al., 2014; Kukla et al., 2018). This technique has been reported to result in extensive soil degradation (Are et al., 2009) and loss of soil organic matter (Ogle et al., 2005; Huon et al., 2013), probably related to the extensive mechanical removal of vegetation, the disturbance of soil surface, and the fire effects. In contrast to these reports, swidden agriculture in rainforests in Papua New Guinea, including slash-and-burn, is extremely sustainable (for several millennia) and has little effects on soil characteristics (Kukla et al., 2018). This is probably because of a small area where swidden agriculture is conducted, a low intensity of fire (due to high moisture and frequent rainfall in the rainforests), absence of mechanical cultivation which would damage soil surface, and the incomplete removal of large woody vegetation (Kukla et al., 2018). Due to its exceptionally sustainable nature, swidden agriculture in these lowland forests requires more attention. The lowland forests in Papua New Guinea are among the world’s largest undisturbed tropical forests (Whitfeld et al., 2012) and exhibit high biological diversity and endemism (Whitfeld et al., 2014). The sustainability and restoration of this ecosystem after swidden agriculture are certainly dependent on soil microbial communities that are supposed to play pivotal roles in ecosystem functioning (e.g., decomposition of substrates, fluxes of energy and materials) (Harris, 2009; Wagg et al., 2014). Therefore, exploring soil microbial community dynamics and their potential driving forces along the spontaneous restoration of this ecosystem, provides a unique opportunity to understand microbial ecological roles in the sustainable reutilization of soils. Although the spontaneous restoration of this ecosystem has been the subject of plant and soil ecology studies (Edwards and Grubb, 1977; Whitfeld et al., 2012; Whitfeld et al., 2014; Kukla et al., 2018), effects of swidden agriculture on soil microbial communities and microbial community succession after the practice in this ecosystem have not been studied. Dynamics of soil microbial community composition are of particular interest, because a recent study finds that nether fungal nor bacterial biomass significantly change across successional stages in this ecosystem (Kukla et al., 2018).

Bacteria and fungi are major components of soil microbial communities and differ in phenotype, nutrition strategy, stress tolerance, ecological functions, and interactions with vegetation; as a consequence, bacterial and fungal communities are driven by different factors and show distinct successional patterns in post-mining fields (Harantová et al., 2017), retreating glacier soils (Brown and Jumpponen, 2014; Franzetti et al., 2020; Garrido-Benavent et al., 2020), forest succession (Chai et al., 2019) and reclaimed mined soils (Sun et al., 2017). Despite the different successional patterns, the dynamics of bacterial and fungal communities are closely related to the development of local ecosystems (Brown and Jumpponen, 2014; Harantová et al., 2017; Sun et al., 2017). Therefore, investigation of both bacterial and fungal communities is necessary, especially in successional soils.

Microbial community diversity is typically attributed to a large number of rare taxa and a small number of highly abundant taxa (dominant taxa) (Jousset et al., 2017; Mo et al., 2018). Because the abundance of microorganisms reflects their adaptabilities and nutritional strategies (Rivett and Bell, 2018), taxa that differ in abundances in a community are probably affected by different biotic/abiotic factors and will have different dynamics during ecological succession. In studies of microbial communities, dominant taxa often receive the most attention, while rare taxa are frequently ignored, because the former are thought to be essential for ecosystem functioning (Lynch and Neufeld, 2015; Mo et al., 2018). However, evidence increasingly indicates that rare taxa are important to ecosystem functioning and stability (Lynch and Neufeld, 2015; Jousset et al., 2017). In addition, researchers have recently suggested that rare and dominant taxa occupy different niches and contribute differently to ecosystem functioning (Jousset et al., 2017). For example, rare taxa have been found to greatly affect nutrient cycling (Pester et al., 2010) and plant biomass accumulation (Hol et al., 2010), and to help to insure system stability (Yachi and Loreau, 1999). These results indicate that consideration of both rare and abundant taxa is required to understand the development of microbial community structure and function during ecological succession.

In this study, three hypotheses are made: (i) distinct successional patterns are observed between bacterial and fungal communities, as well as between their abundant and between rare subcommunities during the restoration of lowland forests following swidden agriculture; (ii) successional dynamics of fungal communities than bacterial communities are more affected by spatial factors; (iii) rare subcommunities of both bacteria and fungi show greater variation than abundant subcommunities during succession. To test these hypotheses, the high-throughput sequencing technology was used to investigate the changes in the bacterial and fungal communities during the secondary succession in swidden agricultural sites in the lowland forests of Papua New Guinea.

## Materials and Methods

### Study Site, Sample Collection, and Processing

The study was conducted near the village of Wanang (145°5′32″ E, 5°14′26″ S), in Madang Province, Papua New Guinea. In this area, the altitude is 100–200 m a.s.l., the mean annual temperature is 26°C, and the mean annual precipitation is 3,500 mm (McAlpine, 1983; Whitfeld et al., 2014). This area is a large (>100,000 ha) continuous lowland rainforest on latosols (Wood, 1982), with a low human population density (<10 persons per km^2^), who mainly live via swidden agriculture. To conduct swidden agriculture in this area, farmers fell small patches of primary or secondary forests. Vegetation burning is very difficult due to high moisture and frequent rainfall, so only dry foliage and smaller branches are usually burned, while trunks and larger branches remain. After a low intensity of burning that releases nutrients from the aboveground vegetation into the soil, farmers finally cultivate the patch for a short term. The resulting “gardens” are subsequently abandoned for spontaneous re-establishment of forests. Specifically, in active gardens, a low intensity cultivation of four crops (e.g., banana, taro, sweet potato, and sugar cane) is conducted; after 2–3 years, these gardens are abandoned, and secondary succession occurs without anthropogenic disturbance until primary forests regrow. Subsequently, active gardens are re-established by swidden agriculture. The chronosequence plots used in this study contained active gardens (Active, current utilization; plant richness (number of species) = 4 including banana, taro, sweet potato, and sugar cane), old gardens (Short term, 5–10 years after abandonment; plant richness = 49 ± 14 per 0.25 hectare, dominated by *Trichospermum pleiostigma*, *Ficus pungens*, and *Macaranga tanarius*), secondary forest (Mid-term, 20–40 years after abandonment; plant richness = 52 ± 16, dominated by *Trichospermum pleiostigma*, *Ficus pungens*, and *Macaranga tanarius*), and primary forest (Primary forest, at least 60 years after abandonment; plant richness = 104 ± 10, dominated by *Pometia pinnata*, *Horsfieldia basifissa*, and *Teijsmanniodendron bogoriense*) (Supplementary Table 1 and Supplementary Figure 1; Whitfeld et al., 2014; Kukla et al., 2018). The number of years since these plots had been abandoned was estimated based on aerial photography and information provided by landowners (Whitfeld et al., 2014; Kukla et al., 2018). Although it is a cycle of “primary forest—active gardens—old gardens—secondary forest—primary forest,” active gardens were used as the starting-point of a chronosequence in this study. This is because this study aimed to explore soil microbial community succession along the ecosystem restoration after swidden agriculture, and the chronosequence (soil age since abandonment) of these study plots is well established.

In June 2013, 12 plots were designated, with three replicate plots for each stage (active gardens, old gardens, secondary forest and primary forest). Individual replicate plots of the same stage were separated by at least several hundred meters and usually by several kilometers (Supplementary Table 1 and Supplementary Figure 1). Individual plots had about 0.1–0.3 ha in area, and soil samples were taken in central part of each plot. In June 2013, soil subsamples were collected at two depths (0–5 and 5–10 cm) by cutting soil bock (5 × 5 × 2 cm), from the four corners of a 20 × 20 m square in middle of each plot; the subsamples were pooled to yield one composite sample per depth per plot. The soil samples were passed through a 2-mm sieve and freeze-dried for DNA isolation and measurement of soil physiochemical properties. Soil total phosphorus (P) and its available forms were determined as previously described (Murphy and Riley, 1962). Soil total carbon (C) and nitrogen (N) were measured with an EA 1108 elemental analyzer. Available forms of calcium (Ca), potassium (K), and magnesium (Mg) were quantified with a Varian SpectrAA-640 atomic absorption spectrophotometer. For soil organic C, the active fractions (including particulate organic matter and dissolved organic C), slow fractions (including C associated with clay and silt and stabilized in aggregates), and passive fractions (oxidation-resistant C) were quantified using a combination of physical and chemical methods (sonication, density fractionation and acid oxidation) (Zimmermann et al., 2007). A soil-water slurry (soil: water, 1:5) was used to measure pH with a pH meter, conductivity with a potentiometric electrode, and NO_3_^–^ concentrations by the colorimetric method. A detailed description of soil properties in these sites can be found in Kukla et al., 2018. Among these soil properties, only the concentrations of available P, Ca, Mg, K, and NO_3_^–^ in soil significantly differed across successional stages (Supplementary Figure 2). DNA was isolated from soil samples according to the modified method of Miller (Sagova-Mareckova et al., 2008). PCR was performed in triplicate for each sample (with the primers gITS7 and ITS4 for the fungal ITS2 region and the primers 515 F and 806 R for bacterial 16S rRNA V4 region) (Supplementary material 1). In total, 48 samples (24 bacterial samples and 24 fungal samples) were prepared for amplicon paired-end sequencing with Illumina MiSeq.

### Data Analysis

QIIME v. 1.9.0 was used for the sequence analyses (Caporaso et al., 2010). Raw sequences were sorted based on their unique barcodes. To obtain clean sequences, low quality sequences, sequences < 150 bp, and chimera were then removed (Edgar and Flyvbjerg, 2015). Clean sequences were clustered into operational taxonomic units (OTUs) at a 97% sequence similarity with the method of CD-HIT (Li and Godzik, 2006). The taxonomic assignments of representative sequences of fungal and bacterial OTUs were based on the UNITE and the Silva databases^1^, respectively. Singletons and sequences that were not assigned to fungi or bacteria were removed. Finally, to minimize the bias of sampling depth on the downstream analyses as well as to make the reads of individual OTUs comparable across samples and successional stages, the clean sequences were standardized to 4,211 reads for each bacterial sample and to 3,863 reads for each fungal sample, based on the minimum read count in bacterial and fungal samples, respectively.

To achieve fungal ecological lifestyles, fungal OTUs were also assigned to ecophysiological groups (trophic categories) using FUNGuild (Nguyen et al., 2016). Fungal OTUs that were not assigned to any ecophysiological groups were grouped into the “Unassigned” category. Alpha diversity indices (Shannon index and Pielou’s evenness) of microbial communities were calculated with the *vegan* package (Oksanen et al., 2013) in R. To reveal the distribution of microbial communities across successional stages and soil depths, non-metric multidimensional scaling (NMDS) ordination with the function *metaMDS*, was conducted based on weighted Bray–Curtis dissimilarity of microbial communities; significant differences of microbial communities across successional stages and soil depths were evaluated by Permutational multivariate analysis of variance (PERMANOVA) test with the function *adonis*. Multiple regression of distance matrices (MRM) based on weighted Bray–Curtis dissimilarity was conducted with the *ecodist* package (Goslee and Urban, 2007) in R. The permutational analysis of multivariate dispersion (PERMDISP) based on the null model (Chase et al., 2011; Zhou et al., 2014) was used to differentiate the observed community beta diversity from the null expectation, so that deterministic and stochastic processes on community assemblies in each successional stage could be evaluated. The null model test was conducted at the pipeline http://ieg.ou.edu/microarray/ by keeping alpha and gamma diversities constant across all the samples and by using the Bray–Curtis dissimilarity of microbial communities. Geographic distance was generated from the GPS coordinates of each sampling site using principal coordinates of neighbor matrices (PCNM) (Legendre et al., 2008). Forward selection was conducted to select edaphic and geographic variables significantly associated (*p* < 0.05) with changes in microbial communities during succession (Blanchet et al., 2008). To explain the changes in microbial communities during succession, these selected variables in forward selection were used in variation partitioning analysis (VPA) and in Mantel and partial Mantel tests based on Spearman’s correlations. Wilcoxon rank sum test, ANOVA permutation tests and Spearman’s correlations were performed in R.

The definitions for abundant and rare OTUs are usually modified based on the specific datasets of microbial communities (Jiao et al., 2017; Yan et al., 2017; Mo et al., 2018; Shu et al., 2018; Jiang et al., 2019). Based on the dataset in this study, we slightly modified the previous definitions (Jiao et al., 2017; Jiang et al., 2019), i.e., we defined locally abundant OTUs as those with relative abundance > 0.5% within a sample, locally rare OTUs as those with relative abundance < 0.05% within a sample, and locally intermediate OTUs as those between locally abundant and locally rare OTUs. At the regional scale, to minimize the artificial change of locally abundant OTUs into regionally intermediate or even rare OTUs because of averaging, regionally abundant/rare OTUs across samples were defined as the union of all locally abundant/rare OTUs in this region. The community consisting of abundant or rare taxa is referred to as the abundant or rare subcommunity, respectively, and the community consisting of all taxa (including abundant, intermediate, and rare taxa) is referred to as the whole community. Based on this definition, there may be some overlaps between regionally abundant and rare subcommunities, namely, some OTUs may be abundant in one sample and rare in another, but it is consistent with the fact that most of OTUs are conditionally abundant or rare (Jiao et al., 2017; Mo et al., 2018; Shu et al., 2018).

## Results

### Diversities of Microbial Communities During Succession

**FIGURE 1:**
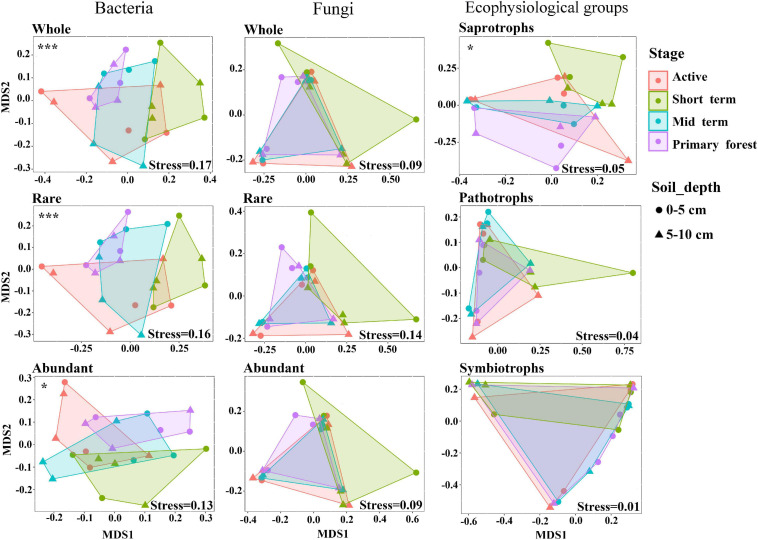
Non-metric multidimensional scaling (NMDS) ordination based on weighted Bray–Curtis dissimilarities of bacterial and fungal communities and fungal ecophysiological groups. Significant differences across successional stages were marked as *p < 0.05 and ***p < 0.001. No significant differences were detected across soil depths.

**FIGURE 2:**
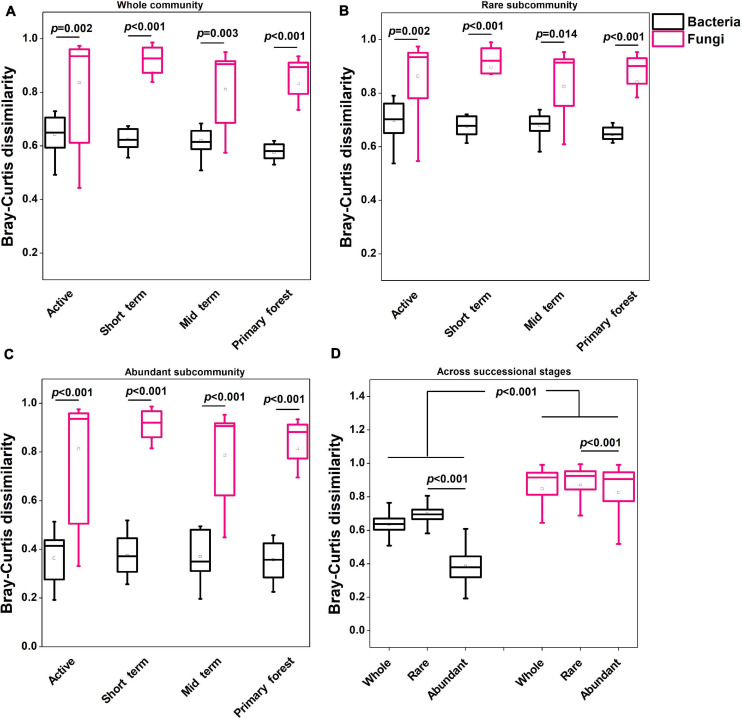
Variation in microbial communities **(A**: Whole community; **B:** Rare subcommunity; **C:** Abundant subcommunity) within each successional stage and across all successional stages **(D)** based on weighted Bray–Curtis dissimilarity. The statistical significance was assessed by the Wilcoxon rank sum test. The square and horizontal black line inside the box represented mean and median, respectively. Data for the regionally abundant and rare OTUs in each stage and across all stages were used.

The alpha diversity in terms of the Shannon index and Pielou’s evenness was higher (*p* < 0.01) in bacterial communities than in fungal communities (Supplementary Figure 3). The NMDS analysis and the PERMANOVA test indicated that only bacterial communities and saprotrophic fungi differed (*p* < 0.05) across successional stages (Figure 1). Neither bacterial nor fungal communities significantly differed across soil depths (Figure 1), so soil depths were unlikely to affect microbial communities. Additionally, neither bacterial nor fungal community dynamics were significantly correlated with plant richness (Supplementary Table 2). The successional dynamics of bacterial communities were more affected by their rare subcommunities than by abundant or intermediate subcommunities, whereas the successional dynamics of fungal communities were most affected by their abundant subcommunities (Supplementary Table 3). For both bacteria and fungi, the contributions of intermediate subcommunities were between those of abundant and rare subcommunities (Supplementary Table 3), which indicated a gradient in the importance of abundance-dependent subcommunities for whole communities. During succession, fungal communities (average Bray–Curtis dissimilarity: 0.84) showed greater (*p* < 0.001) variation than bacterial communities (average Bray–Curtis dissimilarity: 0.55), and this was also evident for abundant and rare subcommunities of fungi and bacteria (Figure 2). For both bacteria and fungi, rare subcommunities (average Bray–Curtis dissimilarity: 0.70 for bacteria, 0.87 for fungi) showed greater variation (*p* < 0.001) than abundant subcommunities (average Bray–Curtis dissimilarity: 0.39 for bacteria, 0.83 for fungi) during succession (Figure 2D).

### Changes in Microbial Community Composition During Succession

**FIGURE 3:**
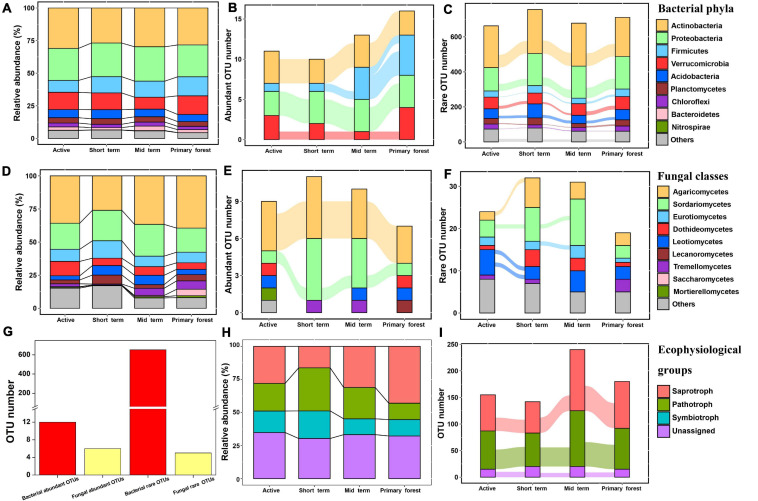
The composition of bacterial communities at the phylum level **(A)**, of fungal communities at the class level **(D)**, and of fungal ecophysiological groups **(H)** in four successional stages at swidden agricultural sites in lowland forests. For abundant OTUs, rare OTUs or fungal ecophysiological OTUs that were present in at least half of the samples in each of two adjacent successional stages, the lines connecting the stages indicated the transfer of those OTUs to the next successional stage: abundant bacterial OTUs **(B)**; abundant fungal OTUs **(E)**; present fungal ecophysiological OTUs **(I)**; rare bacterial OTUs **(C)**; rare fungal OTUs **(F)**; total number of bacterial and fungal abundant/rare OTUs across at least two stages **(G)**. The lines represented the “transfer” of OTUs between stages, and the width of the line indicated the number of OTUs transferred.

At all successional stages, most bacterial OTUs were assigned to Actinobacteria (relative abundance 28–31%), followed by Proteobacteria, Firmicutes, and Verrucomicrobia (Figure 3A). Only the relative abundances of these four phyla plus Bacteroidetes were correlated with edaphic factors (*p* < 0.05), but none of them showed consistent changes during succession (Supplementary Table 4). At the bacterial class level, *Thermoleophilia*, *Alphaproteobacteria*, *Bacilli*, *Actinobacteria*, and *Deltaproteobacteria* were dominant at each successional stage (Supplementary Table 4). At the fungal phylum level, the phyla Ascomycota and Basidiomycota dominated at all stages and together represented about 95% of fungal sequences. At the fungal class level, *Agaricomycetes* was most abundant (relative abundance 26–39%) at each stage, followed by *Sordariomycetes*, *Eurotiomycetes*, and *Dothideomycetes* (Figure 3D). The relative abundances of *Sordariomycetes*, *Eurotiomycetes*, *Leotiomycetes*, and *Tremellomycetes* were correlated with various edaphic factors (Supplementary Table 5). Only the relative abundance of *Tremellomycetes* changed during succession (*p* < 0.05), increasing from the short term stage to the primary forest stage. At the fungal order level, *Hypocreales* was most dominant (relative abundance 12–18%), followed by *Russulales* and *Pleosporales*, in each successional stage (Supplementary Table 5). Compared with other fungal orders, the relative abundance of *Trichosporonales* was more strongly associated with edaphic factors (soil P, Ca, Mg, and K) (*p* < 0.05) and significantly increased with succession. For fungal ecophysiological groups, the relative abundances of pathotrophs and symbiotrophs were higher in early successional stages than in later stages, but the opposite was true for saprotrophs (*p* < 0.05) (Figure 3H). None of the fungal ecophysiological groups changed significantly with succession, but the fungal OTUs assigned to them varied substantially across stages (Figure 3I). In these ecophysiological groups, Ectomycorrhizal, Endophyte, Animal Pathogen, and Plant Pathogen were dominant (Supplementary Table 5).

Both bacterial and fungal communities mainly consisted of rare OTUs rather than abundant OTUs (*p* < 0.01); rare OTUs accounted for the majority of bacterial sequences, whereas abundant OTUs accounted for the majority of fungal sequences (Supplementary Figure 4). The dynamics of abundant/rare OTUs during succession differed between bacteria and fungi (Figure 3). In each successional stage, most of the abundant bacterial OTUs belonged to the phyla Actinobacteria, Proteobacteria, Verrucomicrobia, and Firmicutes (Figure 3B). The following bacterial OTUs were consistently abundant across all successional stages: OTU437 (Order *Gaiellales*), OTU979 (Family *Micromonosporaceae*), and OTU4542 (Order *Acidimicrobiales*) in Actinobacteria, OTU1618 (Genus *Rhodoplanes*), OTU2452 (Order *Rhizobiales*), and OTU3090 (Genus *Rhodoplanes*) in Proteobacteria, and OTU31 (Family *Chthoniobacteraceae*) in Verrucomicrobia. Most of the abundant fungal OTUs belonged to the classes *Agaricomycetes* and *Sordariomycetes* (Figure 3E), and only OTU16 (genus *Russula*; Ectomycorrhizal), OTU38 (genus *Russula*; Ectomycorrhizal), OTU161 (Family *Boletaceae*; Ectomycorrhizal), and OTU476 (genus *Trichoderma*; unassigned ecophysiology) were consistently abundant across all successional stages. Collectively, more bacterial OTUs (*n* = 12) than fungal OTUs (*n* = 6) were consistently abundant across all or several successional stages, and a similar pattern was observed for rare OTUs (bacteria = 653, fungi = 5) (Figure 3G). The bacterial OTUs that were consistently rare across all successional stages mainly belonged to the phyla Actinobacteria, Proteobacteria, Acidobacteria, and Verrucomicrobia (Figure 3C). No fungal OTU was consistently rare across all successional stages (Figure 3F). Across all samples, only bacterial OTU2452 (Order *Rhizobiales*) was consistently locally abundant, but no OTU was consistently locally rare.

### Relationships Between Microbial Communities and Environmental Variables

**FIGURE 4:**
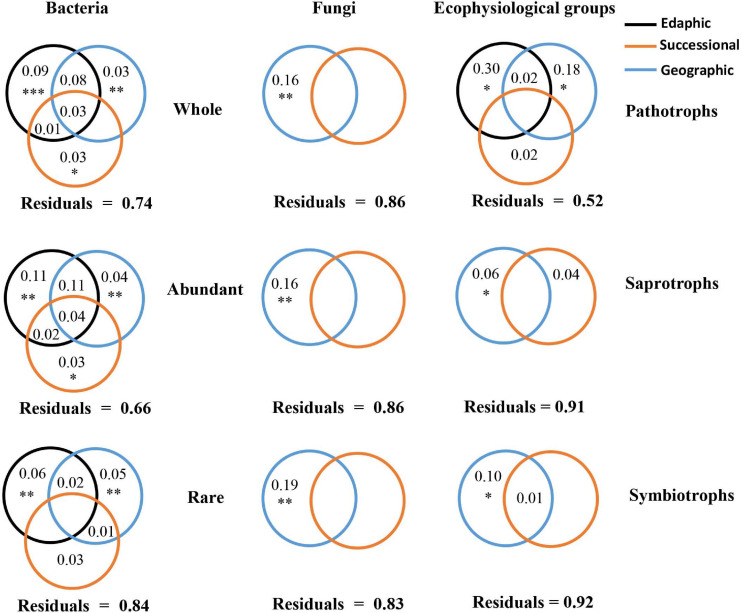
Variation partitioning analysis for the relative contributions of edaphic, geographic, and successional variables to microbial subcommunities and ecophysiological groups. The significance of individual variables was evaluated by ANOVA permutation tests; ^∗^ and ^∗∗^ indicated *p* < 0.05 and < 0.01, respectively. Values < 0 were not shown.

Results of VPA based on forward selection (Supplementary Table 6) showed that the variation in bacterial communities was significantly explained by edaphic variables (9%), geographic distance (3%), and a successional variable (succession stage) (3%), while the variation of fungal communities was significantly explained only by geographic distance (16%) (Figure 4). A similar pattern was observed for both abundant and rare subcommunities, respectively. For fungi, the variation of pathotrophs was explained by edaphic variables (30%) and geographic distance (18%), whereas the variation of both saprotrophs and symbiotrophs was significantly explained only by geographic distance. The VPA results were further supported by the results of Mantel and Partial Mantel (Supplementary Table 7). The significantly positive correlations between average relative abundances and occurrence frequencies of OTUs in both bacteria and fungi (Supplementary Figure 5) further highlighted the effects of geographic distance on rare subcommunities. In each successional stage, the PERMDISP test demonstrated that the bacterial community assemblage differed from the null expectation (*p* < 0.0001), indicating the dominance of deterministic processes driving bacterial community dynamics (Table 1). In contrast, the PERMDISP test indicated that the fungal community dynamics were driven by stochastic processes (Table 1).

**TABLE 1:** Significance test for centroid differences between observed communities in each successional stage and the null model simulations based on Bray-Curtis dissimilarities.

**Community and stage**	**Actual centroid**	**Null centroid**	***F***	***P***
**Bacterial community**				
Active	0.4164	0.5101	56.136	<0.0001
Short term	0.4031	0.5094	62.3473	<0.0001
Mid term	0.3998	0.5135	44.9344	0.0001
Primary forest	0.3723	0.5268	120.978	<0.0001
**Fungal community**				
Active	0.5461	0.5891	0.7033	0.4213
Short term	0.5669	0.5909	0.4719	0.5077
Mid term	0.5349	0.5772	1.4335	0.2588
Primary forest	0.5425	0.5811	2.2156	0.1675

## Discussion

### Successional Patterns of Bacterial and Fungal Communities

Bacterial communities rather than fungal communities clearly changed with succession at swidden agricultural sites. This is consistent with previous findings that fungal communities are more tolerant than bacterial communities to environmental changes that occur during forest restoration (Sun et al., 2017) and bacterial communities are more sensitive than fungal communities to forest restoration process (Banning et al., 2011; Chai et al., 2019). In the current study, however, fungal communities showed higher variation than bacterial communities within successional stages and across stages, respectively (Figure 2). Based on the results of the PERMDISP test, we inferred that the variation in fungal communities within successional stages was caused by stochastic processes, whereas the variation of bacterial communities was caused by deterministic processes, which well corresponded to the significant effect of succession stages on bacterial communities. Remarkably, differences in bacterial and fungal community variations may be also related to different biomarkers used to investigate the two microbial groups. Across stages, bacterial community dynamics were more affected by the development of rare rather than abundant subcommunities, whereas the opposite was true for fungi. In glacial forelands (Yan et al., 2017) and oil-contaminated soils (Jiao et al., 2017), in contrast, abundant bacterial subcommunities were reported to contribute more than rare ones to whole-community dynamics. The difference between our results and previous reports might attribute to different studying environments.

A rare species often shows a more narrow environmental niche than an abundant species (Jousset et al., 2017), which probably contributed to greater variation of rare subcommunities than abundant subcommunities across environmental gradients. We therefore expected greater variation in rare than in abundant subcommunities during succession. This expectation was in line with the results in this study. The succession in this study spanned more than 60 years, during which niche differentiation of microbial communities may have occurred in response to environmental gradients. Niche differentiation may have been greater in the species-rich rare subcommunities than in the species-poor abundant subcommunities, so that the rare subcommunities showed higher variation across successional stages. Additionally, the results of the PERMDISP test and NMDS indicated that deterministic processes and successional stage had greater effects on the rare bacterial subcommunities than on abundant bacterial subcommunities. This indicated a greater degree of niche differentiation during succession in rare than in abundant bacterial subcommunities, which supported the above inference. Thus, rare subcommunities mainly contributed to the niche differentiation of bacterial communities during the succession. Fungal communities, in contrast, tended to show little niche differentiation during succession, because fungal communities were not significantly influenced by successional stage and tended to be assembled stochastically. The higher variation of rare fungal subcommunities than abundant fungal subcommunities might be explained by rare species being more susceptible than abundant ones to local elimination (Melbourne and Hastings, 2008). Remarkably, saprotrophic fungi were significantly affected by successional stage, perhaps because the gradual accumulation of litter during succession (Kukla et al., 2018) increased the availability of nutrients for saprotrophs. The increased abundances of saprotrophic fungi during succession partly supported the above explanation. Collectively, saprotrophic fungi were more sensitive than the whole fungal communities to successional stages. This is also because garden patches are small and experience limited tillage (Kukla et al., 2018), which allows the persistence of the roots of neighboring trees. The roots of these trees would support the persistence of saprotrophic, symbiotic as well as pathogenic fungi.

Consistently abundant bacterial OTUs belonged mainly to the genus *Rhodoplanes*, the order *Rhizobiales* and *Acidimicrobiales*, respectively, which have been found to be abundant in a wide range of environments (Hartmann et al., 2014; Wang et al., 2016; Gkarmiri et al., 2017; Dawkins and Esiobu, 2018) and to possess diverse nutritional strategies (De Mandal et al., 2017). The consistently abundant fungal OTUs belonged mainly to the genus *Russula*, which are symbionts of trees and are therefore abundant in forests (Hartmann et al., 2014). Thus, diverse nutritional strategies and little dependence on vegetation probably caused bacteria to be consistently abundant during succession. The consistent rarity of certain bacterial OTUs during succession in the current study might be explained by narrow niches, abiotic and biotic interactions, or life-history strategies (Jousset et al., 2017; Jia et al., 2018). None of the fungal OTUs, in contrast, was consistently rare across all successional stages, which indicated that fungal rarities were more conditional than bacterial rarities. This can probably be explained by the strong dependence of fungal abundance and presence on vegetation (Urbanova et al., 2015), which changes substantially during succession (Whitfeld et al., 2014), and also by highly spatial variability inside individual stages.

### Ecological Mechanisms Underlying Bacterial and Fungal Succession

Various ecological processes (e.g., environmental selection, geographic effects and stochastic processes) have been invoked to explain the spatiotemporal distributions of microbial communities (Dini-Andreote et al., 2015; Jia et al., 2018; Mo et al., 2018). In this study, geographic distance was found to affect all communities, and especially fungal communities, which were significantly affected only by geographic distance (Figure 4). The effect of geographic distance could be caused by dispersal limitation and/or environmental selection by unmeasured variables which were spatially autocorrelated (Wang et al., 2013; Jiao et al., 2017; Mo et al., 2018). Previous studies have attributed fungal community distribution in bamboo forest soils (Xiao et al., 2018) and in soybean rhizospheres (Zhang et al., 2018) to dispersal limitation. Because fungi are strictly heterotrophic, their distributions depend on the distributions of organic resources (de Boer et al., 2005). Ecological succession on swidden agricultural sites was found to have little effect on soil organic matter in the current study. Collectively, these results indicated that the geographic effects on fungal communities were probably more related to dispersal limitation than unmeasured environmental variables.

Plant community composition has been reported to strongly influence fungal community composition (Urbanova et al., 2015). In the current study, plant richness was not significantly correlated with fungal community dynamics or bacterial community dynamics, indicating little effects of plants on soil microbial community succession. Despite the increase of plant density along restoration of lowland forests (Whitfeld et al., 2014; Kukla et al., 2018), fungal communities did not significantly differ across the chronosequence, which further supported that plant community succession was unlikely to determine fungal community dynamics. This was possibly because the relatively stable soil nutrients (e.g., soil C and N) along succession reduced to some extent the dependence of fungi on plant-derived nutrients. Therefore, geographic effects on fungal communities probably resulted from dispersal limitation rather than from plant community variables in this study. Although no edaphic factors significantly affected fungal communities, the edaphic factors “active and slow fractions of soil organic C” did significantly affect pathotrophic fungi. Perhaps soil organic C indirectly affected pathotrophic fungi by affecting their hosts.

Geographic distance had greater effects on the dynamics of rare bacterial and fungal subcommunities than on abundant bacterial and fungal subcommunities during succession. This finding was not consistent with previous reports from other environments (Wu et al., 2017; Mo et al., 2018), probably because these studies were conducted in ocean but not in successional soils. A high diversity in a microbial community probably implies greater niches differences among taxa within this community, so this community is likely to be determined together by multiple effects rather than a sole effect. Geographic distance factor usually includes mixed effects from multiple factors (e.g., measured and unmeasured environmental factors, and dispersal limitation). Therefore, relative to abundant subcommunities, rare subcommunities consisting of vast diverse species were inferred to be more influenced by geographic distance, which was supported by the results of variation partitioning analysis (Figure 4). Besides being influenced by geographic effects, the succession of bacterial communities was significantly influenced by successional and edaphic factors, which was in line with a report in the restoration of a forest ecosystem (Sun et al., 2017).

## Conclusion

In summary, distinct successional trajectories were observed in whole communities, rare and abundant subcommunities between bacteria and fungi. The successional trajectory of bacterial communities was affected by geographic, edaphic, and successional factors, whereas that of fungal communities was mainly affected by geographic factor. Rare subcommunities of both bacteria and fungi showed greater variation during succession than abundant subcommunities. Rare rather than abundant bacteria mainly drove the bacterial community development and niche differentiation along ecological succession. Consequently, this study has revealed different ecological roles between abundant and rare taxa in the succession of bacterial and fungal communities in swidden agricultural sites in Papua New Guinea, and specifically, rare bacteria (mainly from Actinobacteria, Proteobacteria, Acidobacteria, and Verrucomicrobia) crucially affected bacterial community succession.

## Data Availability Statement

The original sequencing data are available at public database (http://metagenomics.anl.gov/) with dataset ID mgm4829838.3 for bacteria and mgm4829837.3 for fungi.

## Author Contributions

QL involved in data analysis, manuscript writing and revising. PB, LL, and PH involved in reviewing and editing the manuscript. VN involved in sampling, and reviewing and editing the manuscript. JK and LM involved in molecular experiments. RU involved in sampling. JF involved in conceptualizing, reviewing and editing the manuscript. All authors contributed to the article and approved the submitted version.

## Conflict of Interest

The authors declare that the research was conducted in the absence of any commercial or financial relationships that could be construed as a potential conflict of interest.

## Footnotes

1 http://qiime.org/home_static/dataFiles.html
