# Supplementary material for: Dynamics of Soil Bacterial and Fungal Communities During the Secondary Succession Following Swidden Agriculture IN Lowland Forests
Source: Front Microbiol. 2021 Jun 7;12:676251. doi: 10.3389/fmicb.2021.676251 (PMC8215787; doi:10.3389/fmicb.2021.676251)
Supplement: Supplementary file 1 [file Data_Sheet_1.DOCX]

**Supplementary material**

Dynamics of soil bacterial and fungal communities during the secondary succession following swidden agriculture in lowland forests

Qiang Lin^a,f*^, Petr Baldrian^b^, Lingjuan Li^a^, Vojtech Novotny^c,d^, Petr Heděnec^e,g^, Jaroslav Kukla^f^, Ruma Umari^d^, Lenka Meszárošová^b^, Jan Frouz^a,f*^

**Running title:** Soil microbial dynamics during succession

^a^ Biology Centre of the Czech Academy of Sciences, Institute of Soil Biology & SoWa Research Infrastructure, Na Sádkách 7, CZ, 37005, České Budějovice, Czech Republic

^b^ Laboratory of Environmental Microbiology, Institute of Microbiology of the CAS, Vídeňská 1083, 14220 Praha 4, Czech Republic

^c^ Institute of Entomology, Biology Centre of the Czech Academy of Sciences & University of South Bohemia, Branisovska 31, 37005, České Budějovice, Czech Republic

^d^ New Guinea Binatang Research Center, Madang, Papua New Guinea

^e^ Department of Geosciences and Natural Resource Management, Faculty of Science, University of Copenhagen, Rolighedsvej 23, 1958 Frederiksberg C, Denmark

^f^ Institute for environmental studies, Faculty of Science, Charles University, Benátská 2, 12800, Praha 2, Czech Republic

^g^ Engineering Research Center of Soil Remediation of Fujian Province University, College of Resources and Environment, Fujian Agriculture and Forestry University, Fuzhou 350002, China

*******Corresponding author:** Qiang Lin E-mail: **qiangl2019@gmail.com**；Jan Frouz, E-mail: [**frouz@natur.cuni.cz**](mailto:frouz@natur.cuni.cz)

**S1. PCR and sequencing conditions**

Conditions for bacterial amplicons were 94°C for 5 min, 35 cycles at 94°C for 1 min, 50°C for 1 min, 72°C for 1 min, and a final extension at 72°C for 10 min. Conditions for fungal amplicons were 94°C for 5 min, 35 cycles of 94°C for 1 min, 62°C for 1 min, and 72°C for 1 min, and a final extension at 72°C for 10 min (Harantová et al., 2017). PCR products of each sample were pooled and purified using a MinElute Purification Kit (Qiagen). Equimolar mixtures of amplicons were used for library preparation using the TruSeq® DNA PCR-Free LT Kit (Illumina).

**
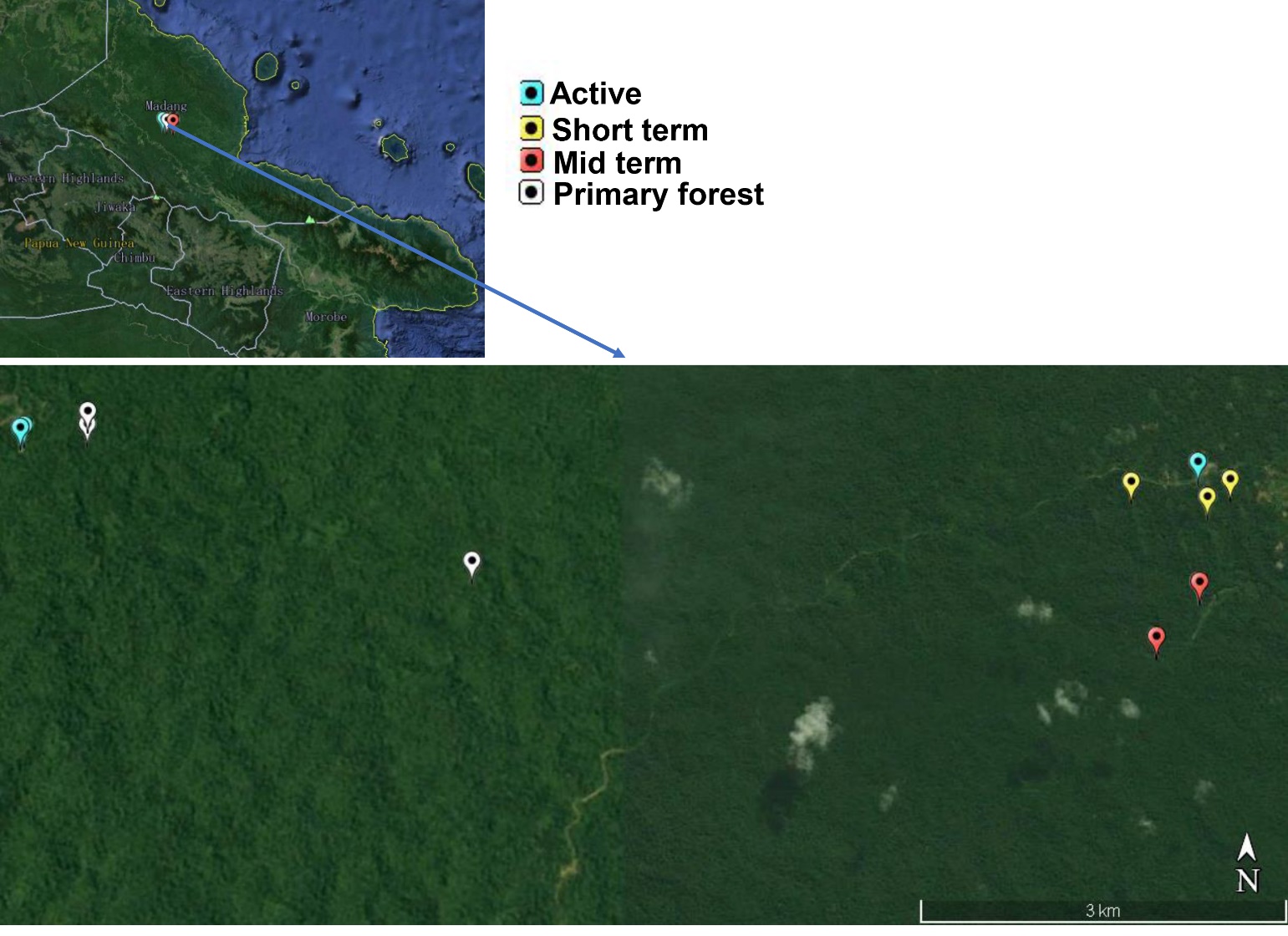
**

**Fig. S1** Map of sampling plots in Papua New Guinea. The map was generated by Google earth (Lisle, 2006).

**
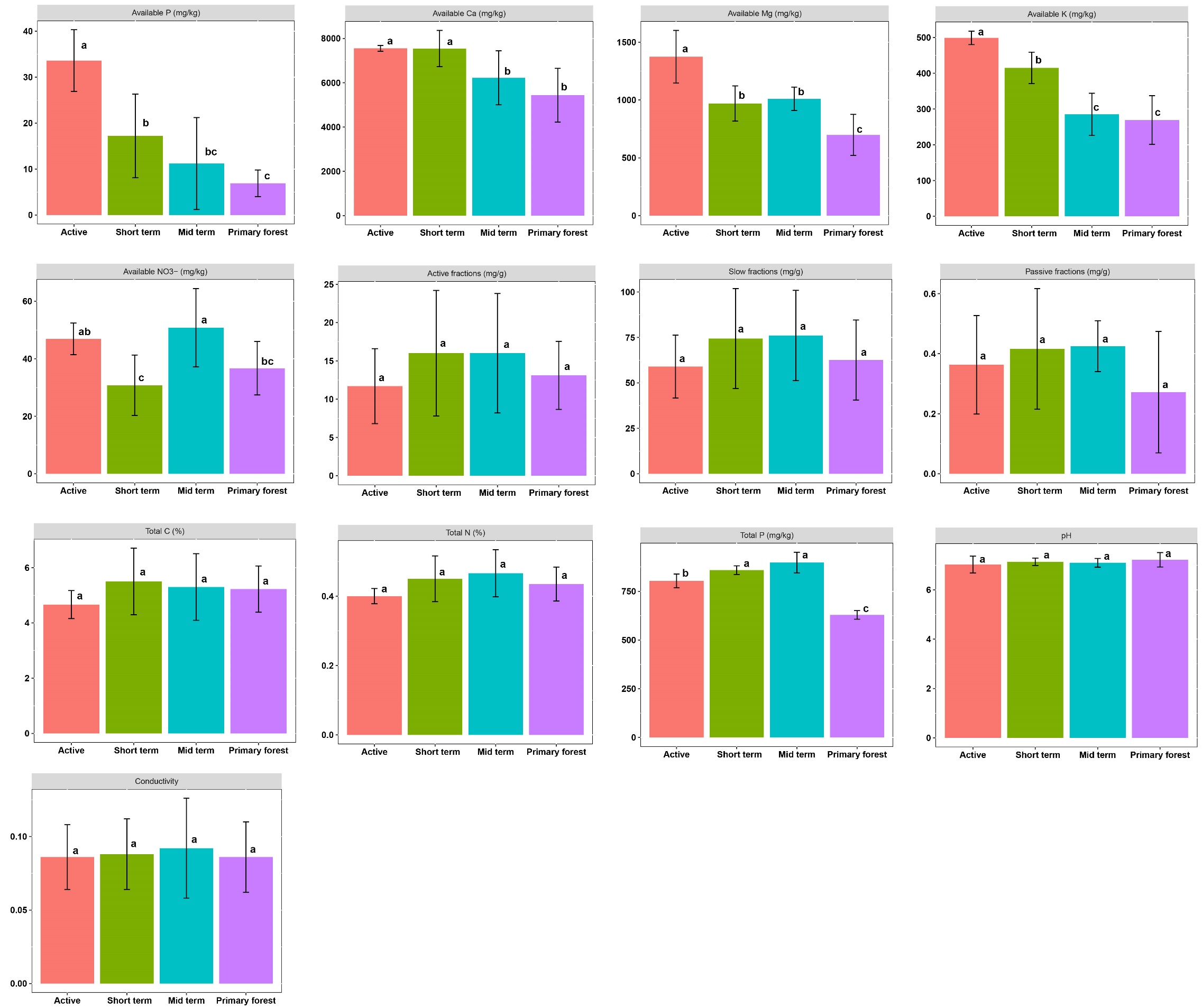
**

**Fig. S2.** Soil properties of the four successional stages. Values were means + SD. Different letters showed significant differences at *p* < 0.05.

**
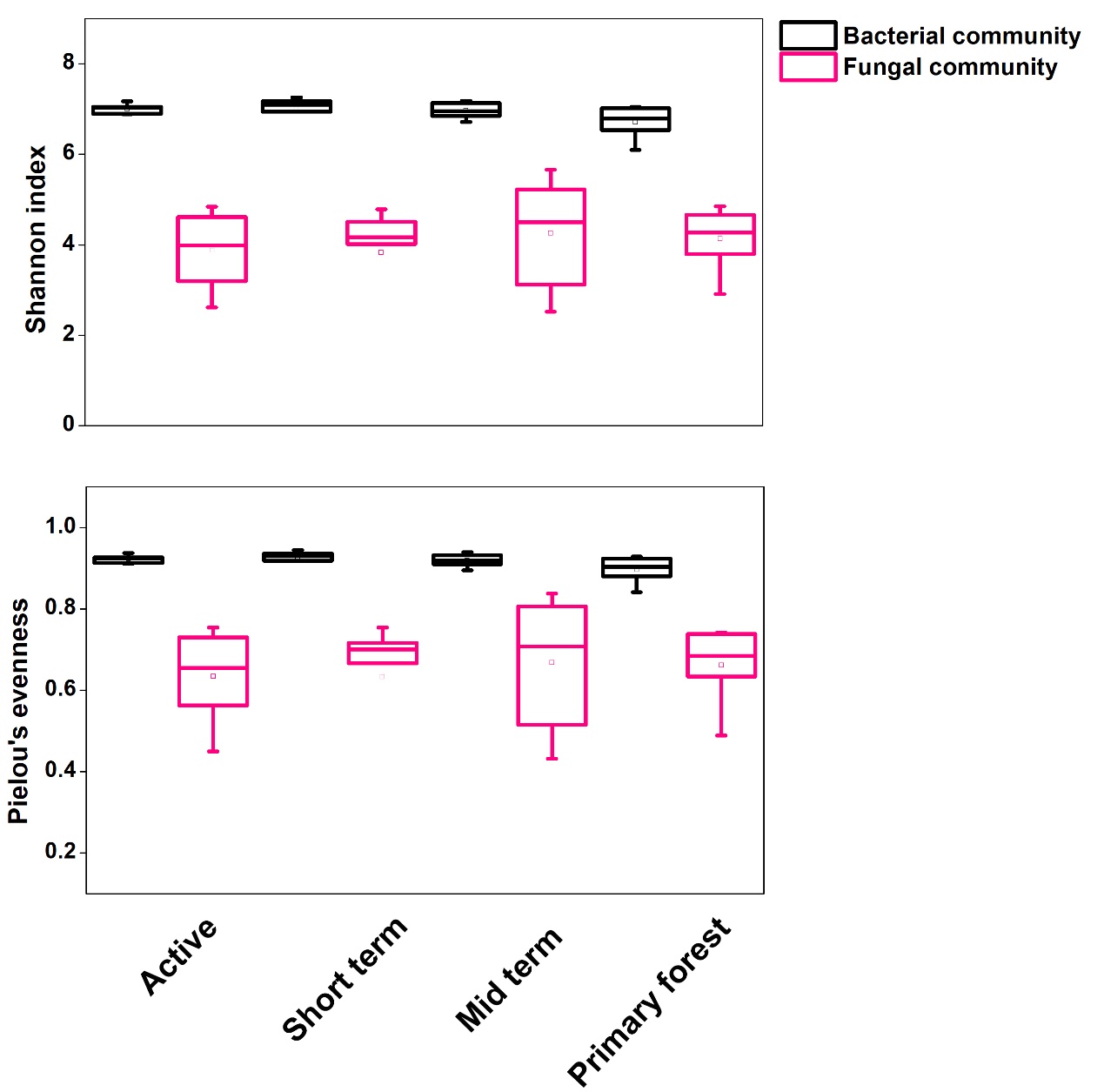
**

**Fig. S3.** Alpha diversities of bacterial and fungal communities in the four successional stages. In each stage, differences between bacterial and fungal communities were significant (*p* < 0.01). Differences among stages were not significant (*p* > 0.05) for each community. The square and horizontal black line inside the box represent mean and median, respectively.

**
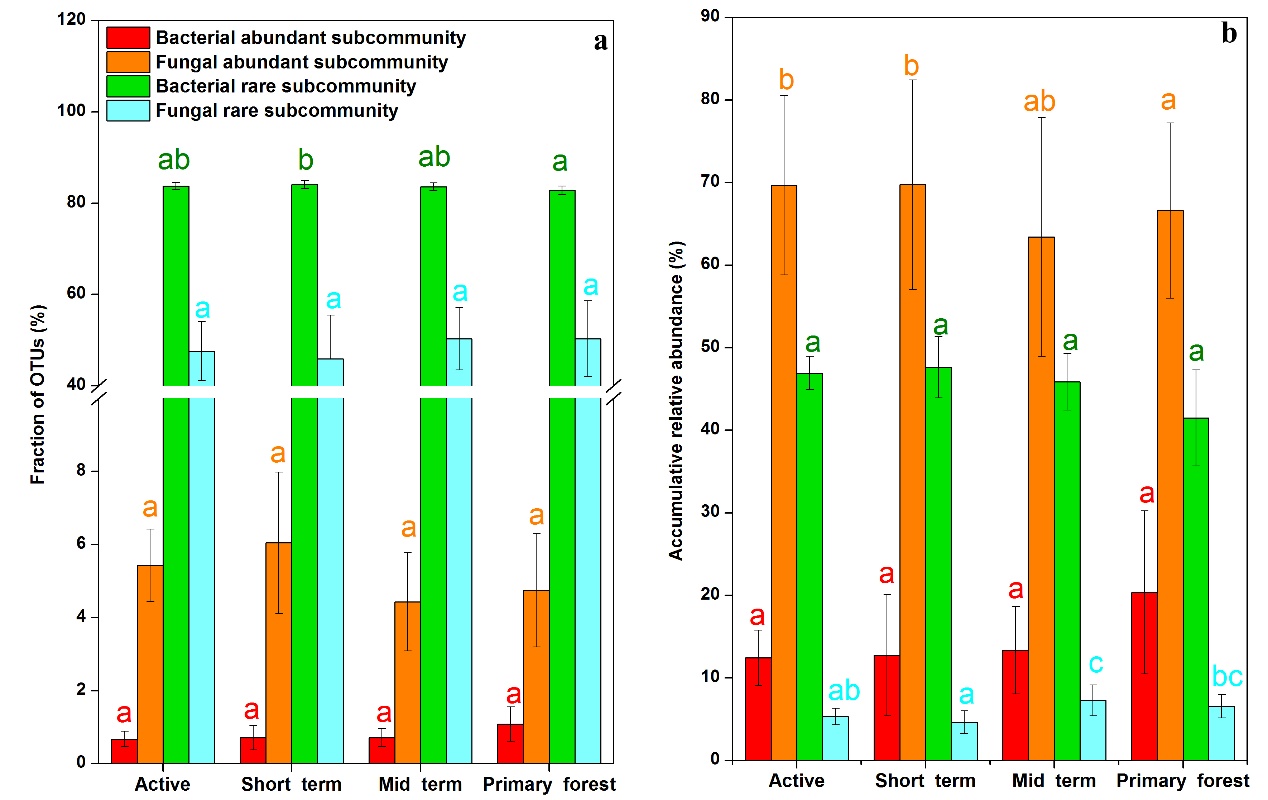
**

**Fig. S4** Percentages of OTUs presented in each subcommunity (a) and corresponding accumulative relative abundances of these OTUs in the four stages of succession (b). Values were means + SD. In each panel and for each successional stage, means with different letters were significantly different at *p* < 0.05. The locally abundant and rare OTUs in each sample were used.

**
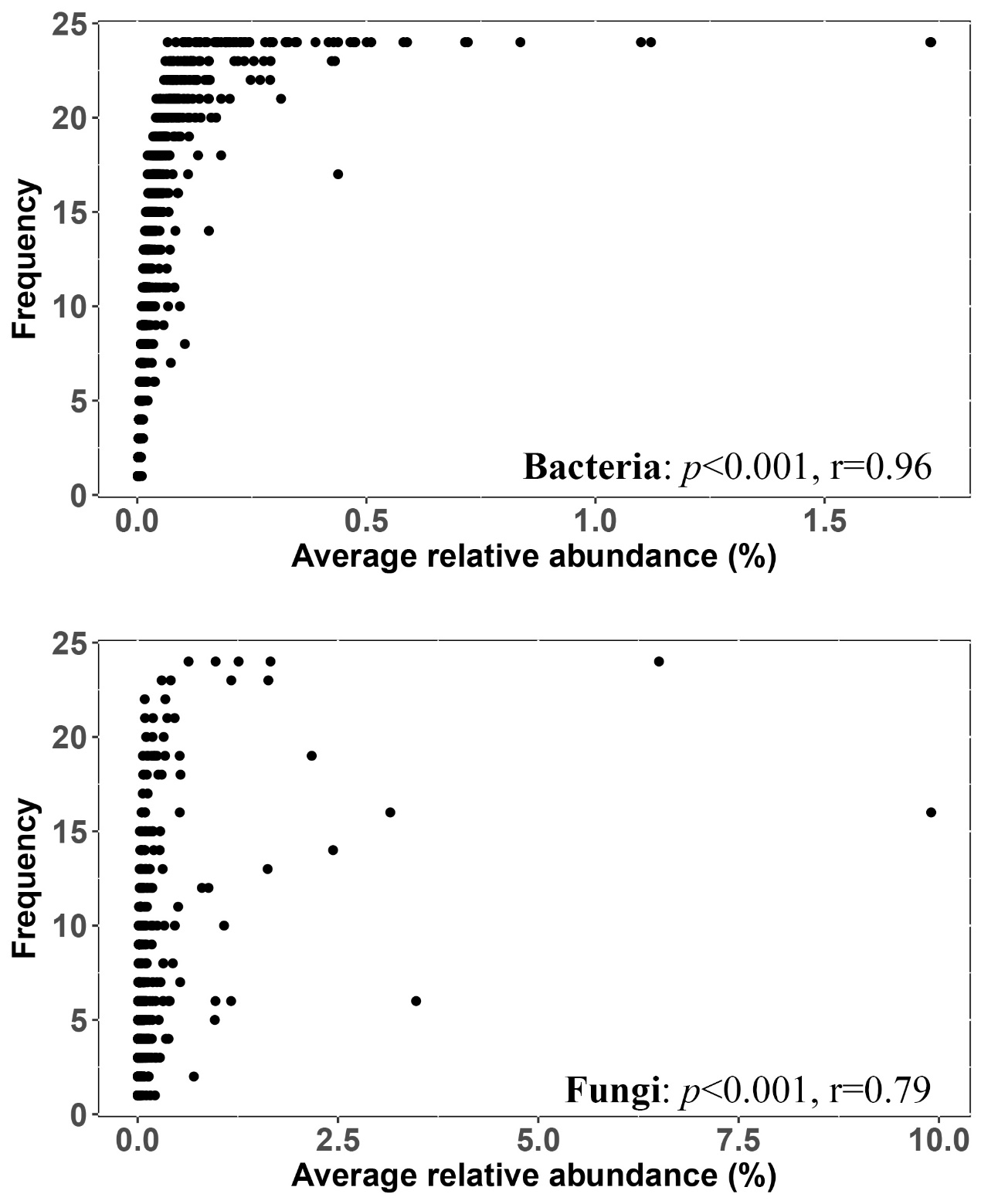
**

**Fig. S5.** Relationships between average abundances and occurrence frequencies of bacterial and fungal OTUs. *P* and r values were evaluated by Spearman’s rank correlation.

**Table S1.** Overview of the study sites (all sites were located in Madang Province, Papua New Guinea, and were sampled at two soil depths in June 2013).

| Site* | Successional age (years) | Geocoordinates |  |
| --- | --- | --- | --- |
| Active1 | 0 | 5°13'39.96"S, 145°4'46.41"E | |
| Active2 | 0 | 5°13'39.36"S, 145°4'47.43"E | |
| Active3 | 0 | 5°13'45.11"S, 145°10'16.30"E | |
| Short term1 | 5-10 | 5°13'51''S, 145°9'57.24''E | |
| Short term2 | 5-10 | 5°13'49.92''S, 145°10'25.02''E | |
| Short term3 | 5-10 | 5°13'54.97"S, 145°10'18.28"E | |
| Mid term1 | 20-30 | 5°14'18.76"S, 145°10'14.38"E | |
| Mid term2 | 20-30 | 5°14'33.95"S, 145°10'1.64"E | |
| Mid term3 | 20-30 | 5°14'18.80"S, 145°10'14.5"E | |
| Primary forest1 | ≥60 | 5°14'15.72''S, 145°6'53.7''E | |
| Primary forest2 | ≥60 | 5°13'38.7''S, 145°5'5.1''E | |
| Primary forest3 | ≥60 | 5°13'34.98''S, 145°5'4.98''E | |

^*^ There were three replicate sites for each of four successional stages.

**Table S2.** Spearman’s correlations between plant richness and microbial community dynamics (indicated by values of MDS1in NMDS).

|  | **Bacteria** |  |  | **Fungi** |  |  |
| --- | --- | --- | --- | --- | --- | --- |
|  | Whole community | Abundant subcommunity | Rare subcommunity | Whole community | Abundant subcommunity | Rare subcommunity |
| Correlation coefficient* | -.041 | .082 | -.064 | -.095 | -.231 | -.125 |

*No significant correlations were observed.

**Table S3.** The contributions of the subcommunity variation to the whole community variation, evaluated by multiple regression of distance matrices based on weighted Bray–Curtis dissimilarities.

| Community | Subcommunity | R^2^ | *P* |
| --- | --- | --- | --- |
| Bacteria | Abundant subcommunity | 0.6707341 | 0.001 |
|  | Intermediate subcommunity | 0.9560225 | 0.001 |
|  | Rare subcommunity | 0.9615986 | 0.001 |
| Fungi | Abundant subcommunity | 0.9904763 | 0.001 |
|  | Intermediate subcommunity | 0.9852312 | 0.001 |
|  | Rare subcommunity | 0.9296202 | 0.001 |

**Table S4.** Relative abundances of bacterial phyla and classes in the four successional stages, and correlations between their relative abundances and environmental variables.

| Taxon | Relative abundance (%) | |  |  | Spearman's correlations | | |  |  |  |  |  |  |  |  |  |  |  |
| --- | --- | --- | --- | --- | --- | --- | --- | --- | --- | --- | --- | --- | --- | --- | --- | --- | --- | --- |
| **Bacterial phylum** | Active | Short term | Mid term | Primary forest | P | Ca | Mg | K | NO_3_^-^ | pH | EC | Active fractions | Slow fractions | Passive fractions | Total C | Total N | Total P | Stage |
| Acidobacteria | 6.25±1.098 | 6.875±1.153 | 5.814±1.901 | 5.466±1.061 | .029 | .040 | .086 | .217 | -.204 | .295 | -.043 | -.025 | -.070 | -.141 | -.157 | -.164 | .102 | -.264 |
| Actinobacteria | 30.986±2.881 | 26.886±2.972 | 29.744±3.691 | 28.342±2.97 | .003 | -.002 | .487^*^ | .115 | .124 | -.343 | .050 | -.108 | -.331 | .067 | -.501^*^ | -.373 | -.079 | -.210 |
| Bacteroidetes | 2.521±0.756 | 1.757±0.436 | 3.428±0.656 | 2.272±0.657 | .010 | -.104 | .174 | -.164 | .638^**^ | .093 | .240 | .365 | .334 | -.120 | .037 | .298 | .172 | .164 |
| Chloroflexi | 2.968±0.806 | 2.581±0.305 | 3.052±0.95 | 2.549±0.579 | -.042 | -.044 | .250 | .084 | .063 | .186 | .147 | .014 | .039 | -.239 | -.250 | -.132 | .042 | -.110 |
| Firmicutes | 9.075±3.823 | 12.44±6.277 | 12.297±6.654 | 14.62±5.569 | -.115 | -.089 | -.414^*^ | -.238 | -.048 | -.113 | -.155 | -.019 | .157 | .188 | .341 | .287 | .026 | .323 |
| Planctomycetes | 4.061±0.535 | 4.282±0.802 | 4.053±0.491 | 3.629±0.401 | .182 | .098 | .370 | .247 | .081 | .241 | .173 | .153 | .007 | .071 | .019 | .077 | .237 | -.285 |
| Proteobacteria | 24.602±3.103 | 25.821±2.721 | 26.375±2.857 | 24.416±2.438 | .062 | -.001 | -.046 | .024 | .039 | .508^*^ | .241 | .100 | .131 | -.016 | .130 | .215 | .272 | -.003 |
| Verrucomicrobia | 13.263±1.902 | 12.871±3.966 | 9.234±1.106 | 14.296±3.587 | .131 | .024 | .080 | .120 | -.028 | -.167 | -.057 | -.051 | -.306 | -.185 | -.172 | -.457^*^ | -.502^*^ | -.094 |
|  | Active | Short term | Mid term | Primary forest | P | Ca | Mg | K | NO_3_^-^ | pH | EC | Active fractions | Slow fractions | Passive fractions | Total C | Total N | Total P | Stage |
| **Bacterial class** |  |  |  |  |  |  |  |  |  |  |  |  |  |  |  |  |  |  |
| Thermoleophilia | 18.313± 1.783 | 14.711± 3.296 | 16.71± 3.59 | 15.4± 3.139 | 0.033 | 0.005 | .503^*^ | 0.144 | 0.148 | -0.048 | 0.14 | -0.104 | -0.289 | -0.086 | -.593^**^ | -.459^*^ | -0.081 | -0.248 |
| Alphaproteobacteria | 15.788± 3.111 | 15.238± 0.86 | 16.865± 2.332 | 16.841± 1.199 | -0.039 | -0.141 | -0.244 | -0.252 | 0.204 | 0.317 | 0.258 | 0.115 | 0.169 | -0.087 | 0.176 | 0.239 | 0.034 | 0.312 |
| Bacilli | 7.31± 3.138 | 11.644± 6.49 | 11.126± 6.222 | 13.54± 5.954 | -0.132 | -0.068 | -.511^*^ | -0.282 | -0.063 | -0.157 | -0.121 | 0.045 | 0.222 | 0.077 | 0.374 | 0.304 | 0.076 | 0.366 |
| Actinobacteria | 7.524± 0.84 | 5.687± 1.26 | 6.59± 1.542 | 5.98± 1.026 | 0.316 | 0.233 | .577^**^ | 0.352 | .447^*^ | -.435^*^ | 0.077 | 0.146 | 0.161 | 0.225 | -0.073 | 0.026 | -0.034 | -0.361 |
| Deltaproteobacteria | 5.43± 1.107 | 6.998± 1.197 | 5.399± 1.201 | 4.805± 0.763 | 0.018 | 0.188 | 0.049 | 0.303 | -0.329 | 0.273 | 0.092 | -0.056 | -0.029 | -0.029 | -0.09 | -0.139 | 0.348 | -0.323 |
| Acidimicrobiia | 3.083± 1.367 | 4.235± 1.194 | 3.914± 0.741 | 4.935± 1.9 | -0.242 | -0.071 | -.425^*^ | -0.225 | -0.291 | -0.238 | -0.218 | -0.03 | 0.078 | 0.127 | 0.33 | 0.307 | 0.067 | 0.326 |
| Planctomycetia | 3.42± 0.599 | 3.15± 0.64 | 3.226± 0.314 | 3.016± 0.33 | 0.346 | 0.079 | .414^*^ | 0.284 | 0.295 | 0.237 | 0.182 | 0.216 | -0.011 | -0.023 | 0.144 | 0.165 | 0.059 | -0.253 |
| Acidobacteria | 2.212± 0.752 | 1.393± 0.489 | 1.389± 0.273 | 1.5± 0.527 | 0.231 | -0.079 | 0.338 | 0.178 | 0.223 | 0.061 | -0.084 | -0.16 | -0.324 | -0.002 | -0.374 | -0.385 | -0.278 | -0.296 |
| Gammaproteobacteria | 3.23± 0.958 | 3.372± 1.389 | 3.982± 0.632 | 2.616± 0.74 | 0.049 | 0.013 | 0.233 | 0.059 | 0.219 | 0.235 | 0.184 | 0.24 | 0.25 | 0.1 | 0.109 | 0.334 | 0.351 | -0.078 |
| Clostridia | 1.678± 1.722 | 0.701± 0.261 | 1.136± 1.018 | 1.005± 0.544 | -0.092 | -0.131 | 0.057 | -0.124 | -0.119 | 0.156 | 0.044 | -0.14 | -.472^*^ | 0.224 | -0.293 | -0.296 | -0.104 | 0.089 |

Only taxa with average relative abundance >1% were shown. * and ** indicated *p* < 0.05 and < 0.01, respectively.

**Table S5.** Relative abundances of fungal classes, orders, and ecophysiological groups in the four successional stages, and correlations between their relative abundances and environmental variables.

| Taxon | Relative abundance | | | | Spearman's correlations | | | | | | | | | | | | | |
| --- | --- | --- | --- | --- | --- | --- | --- | --- | --- | --- | --- | --- | --- | --- | --- | --- | --- | --- |
| **Fungal class** | Active | Short term | Mid term | Primary forest | P | Ca | Mg | K | NO_3_^-^ | pH | EC | Active fractions | Slow fractions | Passive fractions | Total C | Total N | Total P | Stage |
| Agaricomycetes | 35.78 ± 20.063 | 26.033 ± 12.689 | 36.634 ± 21.29 | 39.468 ± 18.828 | 0.085 | -0.098 | 0.076 | 0.013 | 0.165 | 0.34 | 0.061 | 0.117 | -0.275 | 0.101 | -0.106 | -0.067 | -0.163 | 0.054 |
| Sordariomycetes | 19.652 ± 19.568 | 23.035 ± 30.345 | 24.006 ± 18.329 | 18.194 ± 7.565 | -0.127 | -0.168 | -0.281 | -0.128 | 0.038 | 0.167 | -0.071 | -0.101 | 0.251 | -.432* | 0.104 | 0.104 | -0.068 | 0.199 |
| Eurotiomycetes | 9.19 ± 4.681 | 13.146 ± 15.072 | 7.934 ± 5.027 | 7.986 ± 3.519 | 0.348 | 0.202 | 0.203 | 0.159 | 0.355 | 0.122 | 0.236 | .459* | 0.164 | -0.066 | 0.259 | 0.262 | -0.122 | -0.062 |
| Dothideomycetes | 10.846 ± 10.005 | 5.583 ± 3.619 | 6.562 ± 4.666 | 4.953 ± 5.01 | -0.116 | -0.099 | 0.014 | 0.098 | -0.116 | -0.216 | -0.242 | -0.096 | -0.142 | -0.054 | -0.178 | -0.141 | 0.183 | -0.156 |
| Leotiomycetes | 3.167 ± 1.24 | 7.136 ± 6.138 | 7.071 ± 11.444 | 4.012 ± 4.056 | -0.142 | -0.018 | -0.043 | 0.024 | -.438* | -0.152 | -0.26 | -0.353 | -0.335 | 0.063 | -0.382 | -.427* | 0.13 | -0.153 |
| Lecanoromycetes | 2.938 ± 4.775 | 6.8 ± 8.716 | 2.744 ± 5.57 | 4.729 ± 9.973 | -0.14 | -0.013 | -0.173 | -0.108 | -0.346 | -0.029 | -0.245 | -0.289 | -0.105 | 0.057 | -0.058 | -0.079 | 0.129 | 0.003 |
| Tremellomycetes | 2.14 ± 2.43 | 0.733 ± 0.624 | 5.406 ± 9.02 | 6.329 ± 3.719 | -.492* | -.587** | -0.371 | -.494* | -0.131 | 0.113 | -0.355 | -0.185 | -0.286 | -0.083 | -0.291 | -0.214 | -0.284 | .512* |
| Saccharomycetes | 0.263 ± 0.293 | 0.108 ± 0.095 | 0.876 ± 0.832 | 4.867 ± 7.203 | -0.151 | -0.213 | -0.25 | -0.361 | 0.268 | 0.102 | -0.211 | -0.074 | 0.085 | 0.131 | 0.006 | 0.137 | 0.063 | 0.316 |
| Mortierellomycetes | 0.802 ± 0.328 | 0.449 ± 0.291 | 1.005 ± 0.532 | 1.445 ± 1.966 | 0.065 | 0.054 | 0.145 | 0.014 | 0.291 | 0.121 | 0.091 | 0.153 | 0.208 | -0.189 | -0.09 | 0.083 | -0.021 | 0.04 |
| **Fungal order** | Active | Short term | Mid term | Primary forest | P | Ca | Mg | K | NO_3_^-^ | pH | EC | Active fractions | Slow fractions | Passive fractions | Total C | Total N | Total P | Stage |
| Hypocreales | 11.882 ± 14.587 | 18.479 ± 31.566 | 16.559 ± 14.658 | 12.072 ± 6.033 | -0.175 | -0.229 | -0.332 | -0.198 | -0.013 | 0.196 | -0.154 | -0.112 | 0.211 | -0.393 | 0.105 | 0.091 | -0.051 | 0.264 |
| Auriculariales | 16.887 ± 24.91 | 0.155 ± 0.167 | 20.364 ± 28.735 | 14.747 ± 22 | -0.059 | -0.209 | 0.09 | -0.079 | 0.088 | 0.123 | -0.01 | -0.216 | -0.319 | 0.109 | -0.262 | -0.187 | -0.112 | 0.092 |
| Russulales | 11.554 ± 10.876 | 9.712 ± 11.53 | 6.562 ± 4.842 | 5.799 ± 6.36 | 0.05 | -0.021 | 0.027 | 0.101 | 0.1 | 0.258 | -0.164 | 0.186 | 0.02 | -0.059 | 0.1 | 0.172 | 0.042 | -0.059 |
| Pleosporales | 8.974 ± 9.078 | 2.895 ± 1.897 | 4.647 ± 3.866 | 2.899 ± 2.633 | -0.013 | -0.027 | 0.047 | 0.14 | -0.009 | -0.273 | -0.189 | -0.047 | -0.079 | -0.116 | -0.146 | -0.093 | 0.221 | -0.186 |
| Helotiales | 2.382 ± 1.214 | 6.247 ± 5.612 | 6.07 ± 10.421 | 3.184 ± 3.98 | -0.174 | -0.007 | -0.057 | 0.034 | -.462* | -0.124 | -0.275 | -0.391 | -0.303 | 0.039 | -0.365 | -.414* | 0.113 | -0.162 |
| Agaricales | 2.187 ± 1.226 | 4.358 ± 5.407 | 2.131 ± 1.561 | 8.806 ± 3.066 | -0.209 | -0.282 | -0.373 | -0.303 | -0.162 | 0.25 | 0.063 | 0.044 | -0.236 | -0.085 | 0.168 | -0.016 | -.490* | .479* |
| Lecanorales | 2.809 ± 4.748 | 6.623 ± 8.656 | 2.64 ± 5.581 | 4.534 ± 9.908 | -0.108 | -0.015 | -0.074 | -0.082 | -0.218 | -0.045 | -0.188 | -0.223 | -0.157 | 0.072 | -0.143 | -0.141 | 0.189 | -0.073 |
| Eurotiales | 5.842 ± 4.518 | 0.828 ± 0.61 | 5.277 ± 4.899 | 4.025 ± 3.262 | 0.147 | -0.015 | 0.217 | -0.009 | .416* | 0.199 | 0.182 | 0.082 | -0.03 | -0.053 | -0.151 | 0.009 | -0.172 | 0.04 |
| Chaetothyriales | 1.665 ± 0.704 | 8.288 ± 13.062 | 1.726 ± 0.429 | 1.864 ± 1.015 | 0.147 | 0.106 | -0.085 | 0.042 | 0.161 | -0.006 | 0.084 | .499* | 0.161 | -0.02 | 0.289 | 0.277 | 0.167 | 0.003 |
| Sordariales | 5.052 ± 5.535 | 3.18 ± 2.62 | 2.407 ± 0.908 | 2.455 ± 2.25 | -0.14 | -0.041 | -0.072 | 0.042 | -0.141 | 0.043 | 0.002 | -0.097 | -0.13 | -0.285 | -0.239 | -0.227 | 0.155 | -0.092 |
| Trichosporonales | 0.349 ± 0.26 | 0.311 ± 0.411 | 4.677 ± 8.999 | 5.475 ± 3.827 | -.464* | -.660** | -.492* | -.538** | -0.079 | 0.396 | -0.231 | -0.206 | -0.251 | -0.121 | -0.152 | -0.102 | -0.216 | .588** |
| Polyporales | 1.415 ± 1.048 | 1.1 ± 0.731 | 3.296 ± 2.663 | 1.803 ± 1.208 | -0.169 | -0.199 | -0.202 | -0.173 | 0.028 | 0.193 | -0.065 | 0.037 | -0.012 | -0.196 | 0.035 | 0.152 | 0.121 | 0.18 |
| Boletales | 2.127 ± 1.777 | 2.282 ± 1.862 | 1.674 ± 1.053 | 1.355 ± 1.076 | -0.16 | -0.133 | 0.008 | 0.031 | -0.232 | 0.05 | -.421* | -0.174 | -0.293 | 0.062 | -0.227 | -0.203 | 0.06 | -0.14 |
| Saccharomycetales | 0.263 ± 0.293 | 0.108 ± 0.095 | 0.876 ± 0.832 | 4.867 ± 7.203 | -0.151 | -0.213 | -0.25 | -0.361 | 0.268 | 0.102 | -0.211 | -0.074 | 0.085 | 0.131 | 0.006 | 0.137 | 0.063 | 0.316 |
| Xylariales | 1.044 ± 1.021 | 0.505 ± 0.384 | 1.847 ± 1.514 | 2.36 ± 2.356 | -0.133 | -0.218 | -.455* | -0.277 | 0.176 | 0.107 | -0.08 | 0.26 | 0.3 | -0.339 | 0.339 | 0.4 | -0.062 | 0.396 |
| Sebacinales | 0.043 ± 0.044 | 3.46 ± 7.645 | 1.023 ± 2.171 | 0.142 ± 0.203 | -0.391 | -0.289 | 0.023 | -0.237 | -0.222 | -0.114 | -.506* | -0.092 | -0.404 | 0.087 | -0.368 | -0.27 | 0.232 | 0.077 |
| Trechisporales | 0.047 ± 0.035 | 0.035 ± 0.029 | 0.108 ± 0.124 | 3.965 ± 8.589 | -0.213 | -.467* | -0.309 | -.424* | -0.037 | 0.058 | -0.377 | -0.036 | -0.047 | -0.186 | -0.172 | -0.1 | -0.242 | 0.281 |
| Capnodiales | 0.984 ± 0.91 | 1.273 ± 0.988 | 1.04 ± 1.117 | 0.755 ± 0.762 | -0.248 | -0.118 | 0.031 | -0.039 | -0.334 | 0.04 | -0.365 | -0.22 | -0.33 | 0.131 | -0.307 | -0.244 | 0.145 | -0.094 |
| **Fungal ecophysiology^a^** | Active | Short term | Mid term | Primary forest | P | Ca | Mg | K | NO_3_^-^ | pH | EC | Active fractions | Slow fractions | Pasive fractions | Total C | Total N | Total P | Stage |
| Animal Pathogen | 7.628 ±3.196 | 22.841 ±13.593 | 9.177 ±2.562 | 3.849 ±0.485 | 0.021 | 0.241 | -0.02 | 0.124 | 0.009 | -0.235 | 0.056 | 0.222 | 0.324 | 0.115 | 0.338 | 0.367 | 0.297 | -0.118 |
| Plant Pathogen | 5.022 ±2.071 | 2.589 ±0.848 | 7.813 ±2.484 | 3.659 ±0.893 | -0.168 | -0.179 | -0.099 | -0.136 | 0.149 | 0.096 | -0.082 | 0.14 | 0.054 | -0.15 | 0.022 | 0.174 | 0.137 | 0.172 |
| Endophyte | 5.406 ±1.863 | 1.066 ±0.258 | 6.437 ±2.118 | 5.203 ±2.216 | 0.066 | -0.064 | 0.283 | 0.029 | .464* | 0.063 | 0.201 | 0.152 | 0.049 | 0.012 | -0.092 | 0.102 | -0.005 | 0.03 |
| Ectomycorrhizal | 15.446 ±5.606 | 16.736 ±5.931 | 10.769 ±2.55 | 8.948 ±3.042 | 0.058 | 0.093 | 0.115 | 0.182 | -0.049 | 0.118 | -0.167 | 0.121 | -0.117 | -0.07 | -0.05 | -0.025 | 0.129 | -0.188 |
| Arbuscular Mycorrhizal | 0.048 ±0.034 | 0.013 ±0.009 | 0.026 ±0.012 | 0.035 ±0.023 | 0.13 | 0.004 | -0.014 | -0.021 | 0.258 | 0.19 | -0.004 | 0.386 | 0.062 | -.420* | 0.106 | 0.218 | -0.018 | 0.051 |
| Epiphyte | 0.263 ±0.198 | 0.091 ±0.041 | 0.16 ±0.093 | 0.009 ±0.005 | 0.353 | .426* | 0.321 | 0.379 | 0.247 | -0.163 | 0.018 | 0.288 | 0.121 | -0.05 | 0.123 | 0.201 | 0.367 | -0.339 |
| Ericoid Mycorrhizal | 0.125 ±0.061 | 0.138 ±0.064 | 0.181 ±0.038 | 0.164 ±0.044 | -0.374 | -0.247 | -0.145 | -0.272 | -0.196 | 0.138 | -0.108 | -0.337 | -0.361 | .427* | -0.401 | -0.293 | 0.16 | 0.198 |
| Lichenized | 2.753 ±1.903 | 6.45 ±3.75 | 2.835 ±2.366 | 4.608 ±4.344 | -0.164 | -0.102 | -0.164 | -0.12 | -0.102 | -0.221 | -0.191 | -0.187 | -0.102 | -0.074 | -0.168 | -0.138 | 0.13 | -0.003 |

Classes or orders with average relative abundance >1% were shown. * and ** indicated *p* < 0.05 and < 0.01, respectively. ^a^ only the top eight sub-taxa of ecophysiological groups were shown.

**Table S6.** Edaphic and geographic variables significantly associated (*p* < 0.05) with changes of whole communities and subcommunities, respectively, as indicated by forward selection.

| Edaphic variable* | **Bacterial community** | **Whole community** | **Abundant subcommunity** | **Rare subcommunity** |
| --- | --- | --- | --- | --- |
| Mg | R^2^ | 0.1729833 | 0.2147857 | 0.1095996 |
|  | F | 4.601639 | 6.01783 | 2.707986 |
|  | *P* | 0.003 | 0.003 | 0.005 |
| pH | R^2^ | 0.1035055 | 0.1272575 |  |
|  | F | 3.004261 | 4.061678 |  |
|  | *P* | 0.006 | 0.007 |  |
| PCNM2 | R^2^ | 0.1714752 | 0.2193606 | 0.0867239 |
|  | F | 4.55322 | 6.182025 | 2.089101 |
|  | *P* | 0.004 | 0.006 | 0.017 |
| PCNM3 | R^2^ |  |  | 0.0736346 |
|  | F |  |  | 1.84165 |
|  | *P* |  |  | 0.032 |
|  | **Fungal community** | **Whole community** | **Abundant subcommunity** | **Rare subcommunity** |
| PCNM2 | R^2^ | 0.1157273 | 0.1159311 | 0.1229293 |
|  | F | 2.879203 | 2.884939 | 3.083497 |
|  | *P* | 0.014 | 0.018 | 0.028 |
| PCNM3 | R^2^ | 0.1095535 | 0.109761 | 0.1214489 |
|  | F | 2.969623 | 2.976827 | 3.37527 |
|  | *P* | 0.012 | 0.03 | 0.032 |
|  | **Ecophysiological group** | **pathotrophs** | **saprotrophs** | **symbiotrophs** |
| Slow fractions | R^2^ | 0.1279982 |  |  |
|  | F | 3.229304 |  |  |
|  | *P* | 0.036 |  |  |
| Active fractions | R^2^ | 0.2411401 |  |  |
|  | F | 8.027026 |  |  |
|  | *P* | 0.012 |  |  |
| PCNM2 | R^2^ | 0.2022754 |  | 0.1458291 |
|  | F | 5.57844 |  | 3.755971 |
|  | *P* | 0.019 |  | 0.03 |
| PCNM5 | R^2^ |  | 0.0942861 |  |
|  | F |  | 2.29023 |  |
|  | *P* |  | 0.033 |  |

*only significant variables were shown.

**Table S7.** Mantel and Partial Mantel tests for Spearman’s correlations between microbial community variation and variation of edaphic, geographic, and temporal variables with 999 permutations.

| **Bacterial community** | | **Whole community** | | **Abundant subcommunity** | | **Rare subcommunity** | |
| --- | --- | --- | --- | --- | --- | --- | --- |
|  | Controlling for | *P* | r | *P* | r | *P* | r |
| Edaphic |  | 0.163 | 0.09392 | 0.186 | 0.08657 | 0.084 | 0.1283 |
| Geographic | | **0.024** | 0.127 | 0.118 | 0.07956 | **0.023** | 0.1354 |
| Successional |  | 0.213 | 0.06128 | 0.221 | 0.05948 | 0.053 | 0.1163 |
| Edaphic | Geographic | 0.239 | 0.0728 | 0.239 | 0.06413 | 0.125 | 0.1131 |
| Edaphic | Successional | 0.219 | 0.07911 | 0.251 | 0.06352 | 0.167 | 0.08989 |
| Geographic | Edaphic | **0.036** | 0.1125 | 0.162 | 0.05426 | **0.031** | 0.1211 |
| Geographic | Successional | **0.045** | 0.1144 | 0.13 | 0.06522 | 0.056 | 0.107 |
| Successional | Edaphic | 0.294 | 0.03436 | 0.436 | 0.00798 | 0.163 | 0.07147 |
| Successional | Geographic | 0.344 | 0.02571 | 0.263 | 0.03814 | 0.142 | 0.08119 |
| **Fungal community** | | **Whole community** | | **Abundant subcommunity** | | **Rare subcommunity** | |
|  | Controlling for | *P* | r | *P* | r | *P* | r |
| Geographic | | 0.08 | 0.08834 | 0.061 | 0.09293 | 0.058 | 0.09803 |
| Successional |  | 0.568 | -0.01943 | 0.583 | -0.02508 | 0.361 | 0.0151 |
| Geographic | Successional | 0.066 | 0.09823 | 0.058 | 0.1048 | 0.071 | 0.09787 |
| Successional | Geographic | 0.679 | -0.04728 | 0.73 | -0.05463 | 0.529 | -0.01402 |
| **Ecophysiological groups** | | **Pathotrophs** |  | **Saprotrophs** |  | **Symbiotrophs** | |
|  | Controlling for | *P* | r | *P* | r | *P* | r |
| Edaphic |  | **0.034** | 0.18 |  |  |  |  |
| Geographic | | **0.039** | 0.211 | 0.291 | 0.04764 | **0.029** | 0.2227 |
| Successional |  | 0.918 | -0.1021 | **0.027** | 0.1628 | 0.769 | -0.04822 |
| Edaphic | Geographic | **0.03** | 0.193 |  |  |  |  |
| Edaphic | Successional | 0.0502 | 0.1726 |  |  |  |  |
| Geographic | Edaphic | **0.029** | 0.222 |  |  |  |  |
| Geographic | Successional | **0.023** | 0.2385 | 0.298 | 0.05192 | **0.015** | 0.2381 |
| Successional | Edaphic | 0.885 | -0.08813 |  |  |  |  |
| Successional | Geographic | 0.987 | -0.1524 | 0.018 | 0.1641 | 0.944 | -0.09886 |

Microbial community variation was evaluated by Bray–Curtis dissimilarities, and the variation of other variables were evaluated by Euclidean distances.

**Reference**

Harantová, L., Mudrák, O., Kohout, P., Elhottová, D., Frouz, J., & Baldrian, P. (2017). Development of microbial community during primary succession in areas degraded by mining activities. Land Degradation & Development. 28, 2574-2584. doi:10.1002/ldr.2817

Lisle, R. J. (2006). Google Earth: a new geological resource. Geology today. 22, 29-32.
